# The Roles of Insulin-Like Growth Factors in Mesenchymal Stem Cell Niche

**DOI:** 10.1155/2017/9453108

**Published:** 2017-02-16

**Authors:** Amer Youssef, Doaa Aboalola, Victor K. M. Han

**Affiliations:** ^1^Department of Biochemistry, Western University, London, ON, Canada; ^2^Department of Paediatrics, Schulich School of Medicine and Dentistry, Western University, London, ON, Canada; ^3^Children's Health Research Institute, Western University, London, ON, Canada; ^4^Lawson Health Research Institute, Western University, London, ON, Canada; ^5^Department of Anatomy and Cell Biology, Western University, London, ON, Canada; ^6^King Abdullah International Medical Research Center, National Guard Health Affairs, Jeddah, Saudi Arabia

## Abstract

Many tissues contain adult mesenchymal stem cells (MSCs), which may be used in tissue regeneration therapies. However, the MSC availability in most tissues is limited which demands expansion in vitro following isolation. Like many developing cells, the state of MSCs is affected by the surrounding microenvironment, and mimicking this natural microenvironment that supports multipotent or differentiated state in vivo is essential to understand for the successful use of MSC in regenerative therapies. Many researchers are, therefore, optimizing cell culture conditions in vitro by altering growth factors, extracellular matrices, chemicals, oxygen tension, and surrounding pH to enhance stem cells self-renewal or differentiation. Insulin-like growth factors (IGFs) system has been demonstrated to play an important role in stem cell biology to either promote proliferation and self-renewal or enhance differentiation onset and outcome, depending on the cell culture conditions. In this review, we will describe the importance of IGFs, IGF-1 and IGF-2, in development and in the MSC niche and how they affect the pluripotency or differentiation towards multiple lineages of the three germ layers.

## 1. Introduction

Currently, many diseases associated with organ failure and degeneration which are untreatable by pharmaceuticals or organ replacement have seen the promise in cell-replacement and tissue regeneration therapies [1]. Such diseases include endocrine (diabetes), neurodegenerative diseases (Parkinson's, Alzheimer's, and Huntington's), and cardiovascular diseases (myocardial infarction and peripheral vascular ischemia) and injuries or chronic conditions in the cornea, skeletal muscle, skin, joints, and bones [2]. Stem cells have the potential for tissue/organ repair, replacement of dying cells, and promoting the survival of damaged tissues [3]. In addition, with the ability to generate induced pluripotent stem cells from the recipient's own somatic cells [4–6] and the availability of new gene editing technologies (e.g., CRISPR-Cas9 and TALEN) [7, 8], the use of stem cells in many genetic and acquired diseases is closer to reality in the near future.

Adult mesenchymal stem cells (MSCs) are multipotent cells with a defined capacity for self-renewal and differentiation into cell types of all three germ layers depending on their origin. Unlike embryonic stem cells, MSCs have less ethical controversies and lower tumorigenicity; however, they have restricted differentiation potential [9]. Recent research has also demonstrated a transdifferentiation ability of MSCs from cells of one germ layer to another [10]. In addition, MSCs have an immunomodulatory effect to reduce an immune response and are able to be engrafted successfully in therapy resistant graft-versus-host disease [3]. The existence of multipotent stem cells in adult tissues was first described by Till and McCulloch in 1961 [11] and was followed by the isolation of MSCs from bone marrow by Friedenstein in 1968 [12]. Since then, MSCs have been isolated from most mature organs and tissues including skeletal muscle [13], adipose tissue [14], deciduous teeth [15], umbilical cord blood and placenta [16], peripheral blood [17], and brain [18]. Several biological markers characterize MSCs of different origins to be positive for CD73, CD105, CD29, CD44, CD71, CD90, CD106, CD120a, and CD124 and negative for CD117, CD34, CD45, and CD14 [19–21]. MSCs have been demonstrated to differentiate predominantly into mesodermal cells including osteogenic, chondrogenic, adipogenic [22], and endothelial [23] lineages. Also, MSCs can differentiate towards ectodermal lineages including corneal [24, 25] and neuronal cells [26] and also can differentiate towards insulin-producing cells of the endodermal endocrine pancreatic lineage [27].

**Figure 1:**
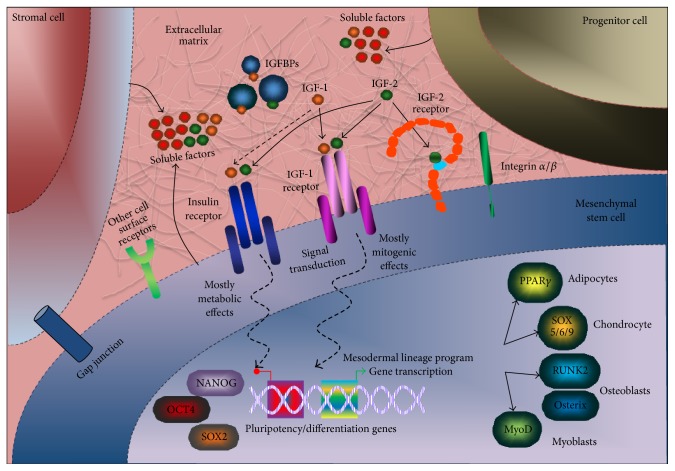
Stem cell niche in vivo. The stem cell niche is a complex compartment surrounding mesenchymal stem cells (MSCs) directing their identity preservation via cellular and acellular components. Various clues and signals are exchanged between MSCs, stromal cells, and progenitor cells and the extracellular matrix containing different soluble factors, oxygen tension, and pH. Therefore, MSC niche manipulates the stemness state of MSCs following growth and regeneration demand. IGFs can signal* via* paracrine/autocrine (produced locally by the tissue) or endocrine (delivered by blood supply) routes to interact with IGF-1 receptor, IGF-2 receptor, or the insulin receptor on MSCs and other cells. IGFBPs (extracellular and/or intracellular actions) can modify IGF actions and affect their stability and degradation. Other receptors and integrins are expressed in MSCs and can be affected by extracellular microenvironment. MSC differentiation occurs by signal transduction which controls the shutdown of pluripotency-associated genes, such as OCT4, SOX2, and NANOG, for the upregulation of differentiation genes. For example, MSCs can give rise to all mesodermal lineages depending on the transcription factor expressed to generate adipose, cartilage, bone, and muscle. Also, transdifferentiation of MSCs into endodermal and ectodermal lineages can occur, as reported by in vitro studies.

Stem cell “niche” is a paracellular microenvironment that includes cellular and noncellular components from local and systemic sources that regulate stem cell pluripotency or multipotency, proliferation, differentiation, survival, and localization [28]. Stem cells are maintained by the surrounding microenvironment* via* several cues including physical, structural, neural, humoral, paracrine, autocrine, and metabolic interactions [29]. Therefore, a combination of different microenvironmental signals that are generated during development, healing, or disease states is capable of regulating the tissue regeneration process leading to proliferation, differentiation, or quiescence [30]. In this review, we will focus on the role of insulin-like growth factors (IGFs) in the MSC niche (Figure 1).

## 2. Insulin-Like Growth Factor System: Ligands, Receptors, and Binding Proteins

Insulin-like growth factors (IGFs; IGF-1 and IGF-2) are two small polypeptides (~7 kDa) that regulate survival, self-renewal, and differentiation of many types of cells, including stem cells [31]. In the systemic circulation in postnatal life, IGF-1 levels are regulated by growth hormone (GH), which induces IGF expression and is released by the liver and accounts for 70–90% of circulating IGFs [32]. Knockdown of* Igf-1* from postnatal murine liver accounted for 75% reduction in circulating IGF-1 levels accompanied by a fourfold increase in GH, which can lead to insulin resistance [33]. Even in the absence of hepatic IGF-1, postnatal growth is not affected in mice. This is likely due to extrahepatic tissue expression of IGFs in a paracrine/autocrine fashion, such as in the bone, brain, lung, uterus, ovaries, adipose tissue, and muscle [34]. Under this condition, serum IGF concentrations are regulated by several factors including gender, age, and nutrition status, leading to a variable range of IGF-1 (264–789 ng/mL) and IGF-2 (702–1042 ng/mL) in healthy individuals [35]. In prenatal (embryonic/fetal) life, the regulation of the synthesis of IGF-1 and IGF-2 by many different organs and tissues is less well understood. Most likely, the synthesis is regulated by local (paracrine) factors and cues such as nutrient status, oxygen tension, biochemistry, extracellular matrix, and other growth factors in addition to endocrine factors. Importantly, the IGFs are synthesized as required by the developmental and physiological cues within the extracellular and intracellular environment. It is likely that the fate of mesenchymal stem cells which reside in the paracellular niches in adult tissues is regulated by the tissue microenvironment.

At the molecular level, IGF-1 shares more than 60% sequence homology with IGF-2 and 50% with proinsulin [42, 43]. IGFs signal mainly* via* the IGF-1 receptor (IGF-1R), which has the highest binding affinity (Kd of 1 nM) towards IGF-1, followed by 10-fold lower affinity to IGF-2 [44]. IGF-1R is a receptor-tyrosine-kinase (RTK) which shares a structural homology domain with the insulin receptor (IR). In turn, IR is expressed in two isoforms, IR-A and IR-B, and can form hybrid receptors (HR-A and HR-B) with the IGF-1R, which binds to both IGFs with variable affinities [45]. Unlike IGF-1, IGF-2 binds to its specific receptor, IGF-2R, and, similar to insulin, it can bind to IR-A [46]. IGF-1R, IR, and HRs are mitogenic RTKs, while IGF-2R is not. Therefore, different receptor and ligand combinations can cause variable signaling outcomes, especially in stem cells. Few studies have been reported on the effects of IGF-1 on the growth, differentiation, and migratory capacity of mesenchymal stem cells [36, 38, 47]; however, the expression of different IGF receptors, insulin receptors, and hybrid receptors and their relative roles in pluripotency and differentiation have not been well studied.

Circulating IGFs are bound to six soluble (~30 kDa) IGF-binding proteins (IGFBPs, 1–6), which determine the bioavailability of free IGF ligand in the extracellular vicinity of the receptors, thus modifying the IGF actions [48]. Under normal physiological conditions, IGFs bind IGFBPs with greater affinity than they bind IGF-1R [49–51]. IGFBPs interaction with IGFs occurs* via* noncovalent binding [52] that protects them from degradation by increasing their half-life [53, 54] and facilitates delivery to specific tissues. Therefore, IGFBPs play an important role in IGF-regulated cell metabolism, development, and growth [55]. In addition, it has become apparent that the IGFBPs can be expressed and maintained within the cellular microenvironment and have additional functions independent of regulating IGFs [56]. The role of IGFBPs in MSC fate is currently being delineated and will be mentioned briefly in this review.

## 3. Insulin-Like Growth Factor System: Signaling Cascades

IGF-1R is a transmembrane tetramer receptor that exists as heterodimers composed of two*α*and*β* hemireceptors linked by disulfide bonds in a*β*–*α*–*α*–*β*structure [57]. Upon ligand binding, the downstream signal of IGF-1R is dependent on the differential phosphorylation pattern of its *β*-subunit and the resultant tyrosine residues available to initiate mitogenic signals, mainly through the phosphoinositide 3-kinase (PI3K), AKT/PKB, and the extracellular signal-regulated kinase (ERK1/2) [55, 58, 59]. In this manner, IGF-1R can induce transcriptional activity to promote survival, self-renewal, and differentiation of MSCs [60, 61].

Upon activation of the extracellular *α* subunits of the IGF-1R, autophosphorylation in the tyrosine residues on*β*-subunits creates high affinity binding sites for signaling adaptor molecules and substrates. For the ERK1/2 signaling pathway, SHC interacts directly with the IGF-1R*β* which recruits GRB2 that interacts with SOS that subsequently activates c-RAS leading to the sequential phosphorylation of RAF, MEK1/2, and then ERK1/2 [42, 62–66]. To activate the PI3K/AKT signaling, p85, the regulatory subunit of PI3K, interacts directly with IGF-1R*β* independent of SHC binding [67]. IRS-1 is a main target of the IGF-1R, implicated in the mitogenic effect of IGF-1R, inhibition of apoptosis, and transformation, whereas its downregulation has been associated with the inhibition of differentiation and the induction of apoptosis [58, 68]. The phosphorylation of IRS-1 amplifies the IGF-1R signaling by indirectly recruiting GRB2 to transduce ERK1/2 signaling [62] or p85 to transduce PI3K signaling [69]. Therefore, surrounding microenvironmental inputs would define stem cell behavior depending on receptor activation and the combination of signaling cascades.

## 4. The Role of IGFs in Growth and Development

During development, circulating IGF levels correlate proportionally with placental and fetal weights, and reduced levels due to poor maternal-nutrition have been suggested to lead to fetal growth restriction [70]. In human pregnancies, IGFs play an early role in promoting proliferation/differentiation and preventing cell apoptosis of various types of placental cells [61]. In mice, knockout of* Igf-1* or* Igf-1r* causes restricted growth (<60% of wild-type) and a premature death of newborn embryos. Most pups with* Igf-1r−/−* are unable to survive due to the lack of functional muscles required for breathing, while some mouse lines with* Igf-1−/−* will survive with deficits in major organs [71–73]. On the other hand,* Igf-2* knockout mice (indistinguishable between homozygous and heterozygous) are viable at 60% birthweight of wild-type [74]. Double mutants for* Igf-1* and* Igf-2* are severely growth-deficient (30% of wild-type) and die shortly after birth of respiratory failure. Although both IGFs have an additive effect in embryonic development, IGF-1 is more important in postnatal growth, while IGF-2 is important for prenatal fetoplacental growth. Hence, IGF-1 and IGF-2 stimulate both proliferation and terminal differentiation of many organs and tissues in developing embryos and adult life. Due to the initial description of* Igf-1*,* Igf-2*, and* Igf-1r* null mice using classic knockout methodology, mice with tissue-specific knockout of these genes have been generated using Cre/loxP conditional knockout strategies with interesting and variable phenotypes [75–78]. The major differences between the models are the tissue/cell-specificity and the timing of the null mutation (prenatal or postnatal). Most of the conditional targeted models are generated with gene targeting after birth to allow examination of the role of* Igf-1* and* Igf-1r* in postnatal development without the ability to discriminate null mutation in stem or somatic cells. In classic knockout models, gene targeting occurs in the embryonic stem cells, allowing us to examine the impact of* Igf* genes in stem cells. However, only very few reports are available investigating the impact of the knockout of* Igf-1* and* Igf-1r* in stem cells. Knockout of* Igf-1r* in adult neural stem cells maintains youthful characteristics of the olfactory bulb neurogenesis within the aging brain by increasing the cumulative neuroblast production and enhancing neuronal integration into the olfactory bulb, suggesting a gain of function during aging [79].

## 5. The Role of IGFs in MSC Multipotency and Self-Renewal

MSCs isolated from different tissues, such as bone marrow, adipose tissue, placental chorionic villi, and fetal membranes, express and secrete IGF-1 and/or IGF-2 in vitro [80–83]. It was shown that ectopic IGF-1 expression in MSCs enhances their proliferation with lower apoptosis [84]. In this study, autocrine IGF-1 levels maintain an elevated baseline activity of ERK1/2 signaling required for enhanced self-renewal (higher OCT4), endodermal (higher CYP51) and mesodermal (higher SM22*α*) potential, but weakened neuronal potential (lower Nestin) [84]. Another growth factor which is basic fibroblast growth factor (bFGF) was shown to be required in maintaining stemness and proliferation in hESCs [31, 85] and in MSCs [80, 86, 87]. Further investigation showed that this bFGF effect was mediated via the IGF system that is upregulated by an autonomous expression of IGF-1R, IGF-1, and IGF-2, as shown in umbilical cord MSCs [86]. Although both IGF-1 and IGF-2 are involved in mediating stem cell fate changes, IGF-2 appears to be more prominent than IGF-1 in promoting MSC pluripotency/self-renewal. In hESCs, one study showed that IGF-2, secreted by spontaneously differentiated autologous fibroblast-like cells in response to bFGF, is required to maintain hESC pluripotency and self-renewal via the signaling of IGF-1R [31]. However, one study showed that hESCs pluripotency/self-renewal maintenance can be independent of IGF-2 secretion only when MSCs are used as a feeder layer [88]. A study, in human dental pulp MSCs (hDSCs), confirmed that IGF-1R is required for MSC multipotency and can be regarded as a selection marker for stemness, just similar to OCT4 and SOX2 [89]. In placental MSCs (PMSCs), IGF-2 is upregulated by low oxygen tension and is required to maintain MSC multipotency [80, 87]. Also, in neural stem cells (NSCs), IGF-2 was shown to play an important role in maintaining self-renewal [90]. In these NSCs, the IGF-2 self-renewal properties were mediated* via* A-isoform of IR (IR-A), independent of IGF-1R or IGF-2R [91]. For a more detailed review on insulin and IGF receptor signaling in neural stem cells, please see Ziegler et al. (2015) [92]. We verified the role of IR-A in PMSC, where we showed an elevated expression level of IR-A versus IR-B; and both IGF-1 and IGF-2 promote increased proliferation and self-renewal with a requirement that both IGF-1R and IR must be present [80]. Although IGF-1R and IR can form hybrid receptors, the role of hybrid receptor in maintaining stem cell fate (ESCs or MSCs) and pluripotency is yet to be confirmed.

## 6. Induction of Same-Origin MSCs towards Different Lineages

In vitro, MSC differentiation can be initiated* via* extrinsic stimulation by growth-factor-mediated differentiation [93] that requires withdrawing maintenance growth factors and adding differentiation promoting growth factors and chemicals [28, 29]. Differentiation factors can include butylated hydroxyanisole and NGF for neuronal differentiation; BMP-12 for tenocyte differentiation; dexamethasone, 3-isobutyl-1-methylxanthine, insulin, and indomethacin for adipogenic differentiation; monothioglycerol, HGF, oncostatin, dexamethasone, FGF4, insulin, transferrin, and selenium for hepatocytic differentiation; b-FGF and VEGF for endothelial differentiation; TGF-*β*1, insulin, transferrin, dexamethasone, and ascorbic acid for chondrogenic differentiation; insulin, transferrin, and selenium for skeletal myogenic differentiation; and dexamethasone, ascorbic acid, and *β*-glycerophosphate for osteogenic differentiation [94–99]. Therefore, the same MSC population in the mesoderm exposed to different extrinsic stimuli can initiate differentiation towards a specific cell type by triggering a tissue-specific transcription factor, such as SOX5/6/9 for chondrocytes, PPAR*γ* for adipocytes, MyoD family for myoblasts, and RUNX2/Osterix for osteoblasts [100]. Moreover, MSCs are shown to transdifferentiate to lineages outside of the mesodermal lineage. In these complex differentiation processes, IGFs are shown to play a role in fine-tuning transcription factor expression levels and activity and defining commitment towards specific lineages from the three germ layers.

## 7. The Role of IGFs in Mesenchymal Stem Cell Fate Specification and Differentiation

### 7.1. Mesodermal Differentiation

#### 7.1.1. Osteogenesis

IGF-1 and IGF-2 are secreted by skeletal bone cells for stimulation of bone formation, growth, and metabolism and prevent apoptosis in a paracrine/autocrine manner [101, 102]. Local overexpression of IGF-1 in osteoblasts can accelerate the rate of bone formation and increase the pace of matrix mineralization, which is dependent on IGF-1R [103]. Decoupling of IGF-1R signaling from IGF-1 is responsible for reduced proliferation/differentiation in primary osteoblast from osteoporosis patients, hence causing bone loss [104]. Under nondifferentiation conditions, IGF-1 transfected human MSCs were able to upregulate expression of various osteoblast genes [105]. For regeneration, bone marrow MSCs (BM-MSCs) secrete IGF-1 and the use of their conditioned media was required to restore alveolar bone regeneration prior to dental implant placement [106]. In dental pulp MSC differentiation, IGF-1 was shown to promote mTOR signaling pathway in order to trigger the expression of RUNX2, OCN, OSX, and COL1 [36]. Also, human deciduous teeth MSCs with high osteopotential express and secrete IGF-2 that is required for differentiation and mineralization [107]. Overall, IGF-1 and IGF-2 play a significant role in MSC osteogenic differentiation and bone health.

#### 7.1.2. Myogenesis

L6E9 cells (a myoblast cell line used in late myogenesis studies), when stimulated with IGF-1, have an initial proliferative response [108]. During rapid cell division, the myogenic regulatory factors are inhibited, whereas, 30 hours later, the mitogenic effect is suppressed and Myogenin expression and activity are increased. Although the downstream factors in IGF-mediated differentiation are still under investigation, IGFs can induce myogenic transcription factors. In contrast, the overexpression of MyoD (a protein that plays a major role in regulating muscle differentiation) in C3H 10T1/2 mouse embryonic fibroblast cells induces IGF-2 expression which in turn activates IGF-1R and its downstream target AKT [109]. Specifically, IGF-2 is required for the recruitment and induction of myogenic promoters and myogenesis [110]. In particular, IGF-2 is required to allow continued recruitment of MyoD-associated proteins at the Myogenin promoter [111]. Moreover, IGF-2 specific binding protein, IGFBP-6, is expressed during embryonic development in many different tissues including the ossifying bones of the cranium, myoblasts, and the motor neurons of the spinal cord [112].

The potential use of MSCs has been investigated in treating muscular injury and myocardial infarction. Rat BM-MSCs have been used as the source of paracrine factors to treat soleus muscle injury [113]. Pretreatment of MSCs with IGF-1 improves MSC healing ability by reducing scar formation, increasing angiogenesis and faster reconstitution of muscle structure, and improving function [113]. In another study, injection of BM-MSCs into the cardiac muscle increased the proliferation and migration and inhibited apoptosis of existing cardiac muscle cells; however, IGF-1 does not induce myocardial differentiation of these MSCs [114]. In addition, transplantation of IGF-1-primed MSCs attenuates cardiac dysfunction, increases the survival of engrafted cells in the ischemic heart, decreases myocardium cell apoptosis, and inhibits the expression of inflammation cytokines such as tumor necrosis factor-alpha (TNF-*α*), interleukin-1*β* (IL-1*β*), and IL-6 [115]. The current research into MSCs and IGF in myogenesis is, therefore, more focused on in vivo muscle repair than on MSC-differentiated myoblast in transplantation.

#### 7.1.3. Adipogenesis

In a comparative study between MSCs from different sources including adipose stem cells (ASCs), bone marrow stem cells (BMSCs), dermal sheath cells (DSCs), and dermal papilla cells (DPCs), IGF-1 had the highest expression and secretion in ASCs compared to the other populations [116]. Also, IGF1 could promote lineage bias and selection for adipogenic progenitor (CD31−/CD34+/CD146−) cells at the expense of the less adipogenic cells (CD31−/CD34+/CD146+) [37]. In this process, IGF-1 attenuates Wnt/*β*-catenin signaling by activating Axin2/PPAR*γ* pathways to promote the selection for (CD31−/CD34+/CD146−) cells [37]. In another study, IGF-1 was shown to alter MSC fate between osteogenic and adipogenic lineages by its ability to bind and form a complex with the acid-labile subunit (ALS) [117]. The loss of this IGF-1/ALS complex shifted differentiation from osteogenesis to adipogenesis [117].

#### 7.1.4. Chondrogenesis

In an intervertebral disc degeneration study, it was shown that IGF-1 and TGF-*β*3 work in synergy to enhance nucleus pulposus-derived mesenchymal stem cells viability, extracellular matrix biosynthesis, and differentiation towards nucleus pulposus cells [118]. Although TGF-*β* signaling is known to be important for chondroinductive differentiation from MSCs, more studies are showing that IGF-1 can regulate MSC chondrogenesis independent of TGF-*β*. In one study, IGF-1 can induce chondrogenic differentiation from adipose-derived MSCs with increased collagen type II, Aggrecan, and SOX9 levels [38]. Similarly, IGF-1 induced the chondrogenic potential of BM-derived MSCs stimulating proliferation, regulating apoptosis, and inducing expression of chondrogenic markers [119]. In this play of IGF-1 in chondrogenesis, IRS-1 localization was induced from being nuclear to being cytoplasmic to shift MSC proliferation to differentiation [120].

### 7.2. Ectodermal Differentiation

#### 7.2.1. Corneal and Neurogenesis

IGF-1 plays an important role in ectodermal lineage differentiation. MSC differentiation towards neural progenitor cells (NPCs) is enhanced by IGF-1 with increased expression of Nestin and the rate of cell proliferation, while it inhibits apoptosis and induces higher terminal differentiation of NPC towards neurons and glial cells [40]. In another study, the administration of IGF-1 alone in addition to corneal extract stimulated differentiation of BM-MSCs into corneal-like cells more efficiently [39]. Mice with reduced IGF-1R expression in the brain in conditional mutant *nes*-*Igf*1*r*^−/Wt^ demonstrate greater neuronal damage following hypoxic-ischemic injury, suggesting the importance of IGF-1R in neuronal cells, including neuronal progenitor and stem cells [121]. IGFBP-2 promotes the keratocyte phenotype of differentiating human corneal fibroblasts from MSCs by increasing the expression of keratocan and ALDH1A1 and decreasing *α*-smooth muscle actin [122]. In addition, IGF-1 overexpressing UC-MSCs are able to differentiate more successfully to neural progenitor cells producing more Pax6-positive cells and Nestin-positive cells and could differentiate into astrocytes with higher efficiency [123].

#### 7.2.2. Epidermal and Dermal Lineages

The role of MSCs and IGFs has been explored in the treatment of skin ulcers, particularly challenging clinical conditions like diabetes [124]. In the developing skin, IGF-1 is expressed in the stratum granulosum, dermal fibroblasts, and the differentiating hair follicles and sebaceous glands [125]. In particular, IGF-1 is strongly expressed in the injury area, where it plays an important role in both epidermal and dermal wound-healing [126]. Recently, BM-MSCs were used in a rodent model of diabetic foot ulceration and were demonstrated to improve wound-healing and to increase the expression of local GFs including IGF-1, EGF, and MMP2 [127].

### 7.3. Endodermal Differentiation

#### 7.3.1. Endocrine Pancreas and Liver

MSC differentiation towards *β*-cells, derived by stepwise media formulation, has not been successful to generate fully functional and glucose-responsive insulin-secreting *β*-cells. However, similar to treating patients with cardiac infarction, MSC transplantation into diabetic patients is being investigated. Umbilical cord MSCs injected in vivo in an induced diabetic rat model were able to prevent hyperglycemic progression and preserve islet size and cellularity; IGF-1 secreted by MSCs was responsible for islet viability and insulin secretion in vitro [128]. Therefore, IGF-1 is a prominent trophic factor in pancreatic islet function and development, which may be required for *β*-cell differentiation in vitro. In hepatocyte differentiation, IGF-1 is expressed and required in the liver during development. It was shown that the addition of IGF-1 to differentiation media induced earlier hepatocyte morphology changes, albumin and AFP expression, glycogen storage, urea production, and albumin secretion [41].

## 8. Crosstalk between the IGF Axis and Other Signaling Pathways in MSC Proliferation and Differentiation

Adult MSCs express different genes associated with both self-renewal and differentiation, including members of the Notch, TGFB, FGF, WNT, IGF, hedgehog families, and G-protein coupled receptor-mediated and cAMP-mediated signaling [129]. Crosstalk between signaling pathways has been shown to be important for stem cells' self-renewal and differentiation; however, specific interactions with the IGF system are still being delineated in MSCs.

As shown in Figure 1, integrins can play an important role in IGF signaling. In particular, IGF-1 directly binds to *α*v*β*3 integrin and induces *α*v*β*3-IGF1-IGF1R ternary complex formation required for phosphorylation, ERK and AKT activation, and cell proliferation [130].

In primary oligodendrocyte precursors, IGF-1 signaling was shown to increase *β*-catenin protein abundance via the IGF-1-induced phosphorylation of AKT and GSK3 required for an increase in cyclin D1 mRNA, proliferation, and survival [131].

To enhance migration and homing after transplantation, IGF-1 upregulates the level of the CXCR4, receptor for the chemokine stromal cell-derived factor-1, and SDF-1, in MSCs and in turn can accelerate migration [132, 133]. In these MSCs, CXCR4 upregulation is mediated by the PI3K/AKT pathway downstream of the activated IGF-1R [133]. In vivo, MSC preconditioning with IGF-1, before administration, was shown to be effective in migration and homing which was required for the restoration of renal function following acute kidney injury [47].

In bone formation and osteoblast differentiation, IGF-2 was shown to potentiate the bone morphogenetic protein-9 (BMP-9), which belongs to the transforming growth factor-*β* (TGF-*β*) superfamily [134]. IGF-2, mediated via the PI3K/AKT, can potentiate BMP-9-induced activity of the early osteogenic marker alkaline phosphatase (ALP) and the expression of later markers such as osteocalcin and osteopontin in MSCs. On the other hand, IGFBP3 and IGFBP4 can inhibit the potentiation effect of IGF-2 on BMP-9-induced ALP activity and matrix mineralization in MSCs [134].

Also, in osteoblast differentiation of BM-MSCs, hedgehog (HH) via Gli2 was shown to increase IGF-2 expression that was acting via the IGF-1R/mTORC2/AKT [135]. IGF-2-mediated AKT activation served as a positive feedback loop for enhanced HH transcriptional output by stabilizing full-length Gli2 due to phosphorylation by AKT. In myogenic differentiation, sonic hedgehog (SHH), member of HH family, was also regarded as positive regulator of IGF-1 signaling in a cooperative additive effect in primary myoblast proliferation and differentiation via the MAPK/ERK and PI3K/AKT pathways [136]. In this process, Smoothened, a SHH effector, can associate with IGF-1R and is required for IGF-1 action via AKT, especially for differentiation.

Crosstalk between the IGF system and other pathways has also been explored in cancer stem cells which may not be dependent on their immediate niche but can give an insight into normal MSCs [137]. In glioma stem cells (GSC), HH via Gli1 upregulated the transcriptional activation of IRS-1 which increased GSC sensitivity to IGF-1 stimulation [138]. In lung adenocarcinoma stem-like cells, IGF-1R-mediated OCT4 expression to form a complex with *β*-catenin and SOX2 was crucial for the self-renewal and oncogenic potential [139]. Other signaling pathways are shown to interact with the IGF system in many cell types that are still to be elucidated in the MSC population and understand their effect in self-renewal and differentiation.

## 9. IGF-Expressing MSCs in Treating Terminal Diseases

Paracrine factors, including IGFs, secreted by MSCs are shown to play a major role in treating organ-failure-causing diseases. IGF-expressing MSCs were shown to enhance proliferation, differentiation, and repair of surrounding tissue in kidney, heart, and pancreas [81–83]. In kidney ischemic-reperfusion injury, physical interaction between MSC and kidney tissue was required to promote kidney repair and not only MSC conditioned media alone [83]. Genetically engineered IGF-1-MSCs were used to treat liver cirrhosis in mice [140]. Transplanted MSC induced higher IGF-1 and HGF expression with lowered TGF-*β*1 levels and less activation of hepatic satellite cells. IGF-1 effect was evident by lowered collagen expression and fibrosis with more parenchymal cell proliferation as indication of liver regeneration. Following myocardial infarction, it was shown that adult human epicardium-derived cells and cardiomyocyte progenitor cells synergistically improve cardiac function, probably instigated by complementary paracrine actions [141]. In fact, cotransplantation of unmodified MSC plus cardiovascular progenitors had elevated expression of factors promoting cardiac repair specifically IGF1 that promoted expression of prosurvival and angiogenesis genes in human cells [142].

In induced diabetes STZ mice, MSC helped to attenuate abnormal function of adipocytes, which are involved in cutaneous wound-healing, by IGF-1 secretion [143]. IGF-1 in these mice helped in activating PI3K/AKT and GLUT4 which improved glucose uptake and insulin sensitivity, therefore improving diabetic wound-healing. In hepatocellular carcinoma (HCC) treatment, fetal human MSCs conditioned media were used to inhibit cell growth [144]. It was discovered that the conditioned media contained high levels of IGFBPs which sequestered IGFs and reduced IGF-1R and AKT activation, leading to cell cycle arrest in HCC. These tumor-specific effects were not observed in matched hepatocytes or patient-derived matched normal tissue. In all these examples, MSCs expressing IGF system components are being used in enhancing tissue repair of failing organs, fighting cancer, and ameliorating diabetes.

## 10. Critical Use of IGF and Insulin in Cell Culture Conditions

Addition of IGFs to differentiation media leads to earlier commitment and higher onset of differentiation in several lineages including endothelial, corneal, neural, chondrocyte, adipocyte, hepatocytes, and osteoblast cells (Table 1). In this context, a typical concentration of 10–100 ng/mL of IGF-1 is sufficient to activate only the IGF-1R and not the IR. On the other hand, not much attention is given to receptor binding affinity of IGF-1R versus IR, when insulin is used in maintenance or differentiation conditions. The inclusion in commercial biological products for stem cell research of nonphysiological concentrations of insulin (0.5, 5, and 10 *μ*g/mL) for MSC differentiation media is 100–1,000x higher than highest insulin concentration in serum [145]. High concentrations of insulin (≥1 *μ*g/mL) not only activate IR but also activate IGF-1R [44, 145]. Therefore, using such high insulin concentrations in defined media, which can be a substitute to IGFs, cannot distinguish whether the effect is mediated via IGF-1R or IR signaling pathways and studies describing the effect of IGFs in growth and/or differentiation of stem cells in “defined medium” should recognize this potential confounding effect.

## 11. Summary and Conclusions

IGFs are among the earliest growth factors to be expressed in a developing embryo as early as in preimplantation embryos and putatively act as autocrine/paracrine factors on many developing cells including stem cells. They form an important component of the stem cell niche. Their expression is ubiquitous in many cell types; however, they are most abundant in the cells and tissues of mesodermal origin. Thus, MSCs are both the source and target of IGFs during development and likely play important roles in the maintenance of pluripotency as well as determining their fate to lineages of all three germ layers. Recent evidence also suggests the potential discriminating roles of IGF-1 and IGF-2 in MSCs and progenitor cells of different tissues. As MSCs are being investigated as being important for cellular replacement and regenerative therapies, delineating the roles of endogenous as well as exogenous IGFs in MSC growth and differentiation will be critical in developing these cellular therapies towards treatment of many degenerative diseases that have no viable therapeutic options at present.

## Figures and Tables

**Table 1:** MSC differentiation protocols with IGFs towards different lineages. Example of MSC differentiation protocols included IGFs in their differentiation media formulations.

MSC population	Differentiation	Protocol	Reference
Human dental pulp stem cells	Osteoblast-like cells	Osteogenic media supplemented with 0.1 *µ*mol/L dexamethasone, 10 mmol/L b-glycerophosphate, 50 *µ*g/mL ascorbic acid, and 100 ng/mL of IGF-1.	[36]
Stromal vascular fraction of adipose tissue, human adipose stem/progenitor cells	Adipocyte-like cells	StemPro® Adipogenesis Differentiation Kit supplemented with 10 ng/mL of IGF-1.	[37]
Human adipose-derived mesenchymal cells	Chondrocyte-like cells	DMEM high glucose supplemented with 1% FBS, 0.1 mM ascorbic acid-2-phosphate, 10^−7^ dexamethasone, 6.25 *μ*g/mL transferrin, 6.25 ng/mL selenous acid, 10 ng/mL recombinant human TGF-*β*1, and 100 ng/mL recombinant human IGF-1.	[38]
Mouse bone marrow mesenchymal stem cells	Corneal-like cells	MSCs were cultured for 3, 7, or 10 days in complete DMEM with 20% extract from the corneas and 20 ng/mL IGF-1.	[39]
Rat bone marrow mesenchymal stem cells	Neural-like cells	Proliferation media: NeuroCult® NS-A proliferation media specific for rat supplemented with 20 ng/mL EGF, 20 ng/mL bFGF, and 100 ng/mL IGF-1. Differentiation media: NeuroCult supplemented with 10 ng/mL PDGF-BB for glial induction or 10 ng/mL rh-BDNF for neuronal differentiation.	[40]
Human bone marrow mesenchymal stem cells	Hepatocyte-like cell	Step 1: DMEM low glucose supplemented with 10% FBS, 20 ng/mL of IGF-I, 20 ng/mL of HGF, and 10^−7^ M dexamethasone for 7 days. Step 2: step 1 media with 10 ng/mL Oncostatin M for 14 days.	[41]

## References

[1] Daley G. Q. (2012). The promise and perils of stem cell therapeutics. *Cell Stem Cell*.

[2] Sánchez A., Schimmang T., García-Sancho J. (2012). Cell and tissue therapy in regenerative medicine. *Advances in Experimental Medicine and Biology*.

[3] Koh M. B. C., Suck G. (2012). Cell therapy: promise fulfilled?. *Biologicals*.

[4] Lowry W. E., Richter L., Yachechko R. (2008). Generation of human induced pluripotent stem cells from dermal fibroblasts. *Proceedings of the National Academy of Sciences of the United States of America*.

[5] Aasen T., Raya A., Barrero M. J. (2008). Efficient and rapid generation of induced pluripotent stem cells from human keratinocytes. *Nature Biotechnology*.

[6] Wang J., Gu Q., Hao J. (2013). Generation of Induced Pluripotent Stem Cells with High Efficiency from Human Umbilical Cord Blood Mononuclear Cells. *Genomics, Proteomics and Bioinformatics*.

[7] Cong L., Ran F. A., Cox D. (2013). Multiplex genome engineering using CRISPR/Cas systems. *Science*.

[8] Reyon D., Tsai S. Q., Khgayter C., Foden J. A., Sander J. D., Joung J. K. (2012). FLASH assembly of TALENs for high-throughput genome editing. *Nature Biotechnology*.

[9] Ding D.-C., Shyu W.-C., Lin S.-Z. (2011). Mesenchymal stem cells. *Cell Transplantation*.

[10] Wakao S., Kuroda Y., Ogura F., Shigemoto T., Dezawa M. (2012). Regenerative effects of mesenchymal stem cells: contribution of muse cells, a novel pluripotent stem cell type that resides in mesenchymal cells. *Cells*.

[11] Till J. E., McCulloch E. A. (2012). A direct measurement of the radiation sensitivity of normal mouse bone marrow cells. *Radiation Research*.

[12] Phinney D. G. (2002). Building a consensus regarding the nature and origin of mesenchymal stem cells. *Journal of cellular biochemistry. Supplement*.

[13] Jankowski R. J., Deasy B. M., Huard J. (2002). Muscle-derived stem cells. *Gene Therapy*.

[14] De Ugarte D. A., Morizono K., Elbarbary A. (2003). Comparison of multi-lineage cells from human adipose tissue and bone marrow. *Cells Tissues Organs*.

[15] Miura M., Gronthos S., Zhao M. (2003). SHED: stem cells from human exfoliated deciduous teeth. *Proceedings of the National Academy of Sciences of the United States of America*.

[16] Mareschi K., Biasin E., Piacibello W., Aglietta M., Madon E., Fagioli F. (2001). Isolation of human mesenchymal stem cells: bone marrow versus umbilical cord blood. *Haematologica*.

[17] Zvaifler N. J., Marinova-Mutafchieva L., Adams G. (2000). Mesenchymal precursor cells in the blood of normal individuals. *Arthritis Research*.

[18] Murrell W., Palmero E., Bianco J. (2013). Expansion of multipotent stem cells from the adult human brain. *PLoS ONE*.

[19] Bianco P., Robey P. G. (2000). Marrow stromal stem cells. *Journal of Clinical Investigation*.

[20] Civin C. I., Trischmann T., Kadan N. S. (1996). Highly purified CD34-positive cells reconstitute hematopoiesis. *Journal of Clinical Oncology*.

[21] Pittenger M. F., Mackay A. M., Beck S. C. (1999). Multilineage potential of adult human mesenchymal stem cells. *Science*.

[22] Abdallah B. M., Kassem M. (2008). Human mesenchymal stem cells: from basic biology to clinical applications. *Gene Therapy*.

[23] Jiang Y., Vaessen B., Lenvik T., Blackstad M., Reyes M., Verfaillie C. M. (2002). Multipotent progenitor cells can be isolated from postnatal murine bone marrow, muscle, and brain. *Experimental Hematology*.

[24] Gu S., Xing C., Han J., Tso M. O. M., Hong J. (2009). Differentiation of rabbit bone marrow mesenchymal stem cells into corneal epithelial cells in vivo and ex vivo. *Molecular Vision*.

[25] Ma Y., Xu Y., Xiao Z. (2006). Reconstruction of chemically burned rat corneal surface by bone marrow-derived human mesenchymal stem cells. *Stem Cells*.

[26] Sanchez-Ramos J., Song S., Cardozo-Pelaez F. (2000). Adult bone marrow stromal cells differentiate into neural cells in vitro. *Experimental Neurology*.

[27] Xie Q.-P., Huang H., Xu B. (2009). Human bone marrow mesenchymal stem cells differentiate into insulin-producing cells upon microenvironmental manipulation in vitro. *Differentiation*.

[28] Jones D. L., Wagers A. J. (2008). No place like home: anatomy and function of the stem cell niche. *Nature Reviews Molecular Cell Biology*.

[29] Scadden D. T. (2006). The stem-cell niche as an entity of action. *Nature*.

[30] Martinez-Agosto J. A., Mikkola H. K. A., Hartenstein V., Banerjee U. (2007). The hematopoietic stem cell and its niche: a comparative view. *Genes & Development*.

[31] Bendall S. C., Stewart M. H., Menendez P. (2007). IGF and FGF cooperatively establish the regulatory stem cell niche of pluripotent human cells in vitro. *Nature*.

[32] Holt R. I. G. (2002). Fetal programming of the growth hormone-insulin-like growth factor axis. *Trends in Endocrinology and Metabolism*.

[33] Yakar S., Liu J.-L., Stannard B. (1999). Normal growth and development in the absence of hepatic insulin-like growth factor. *Proceedings of the National Academy of Sciences of the United States of America*.

[34] Le Roith D., Bondy C., Yakar S., Liu J.-L., Butler A. (2001). The somatomedin hypothesis: 2001. *Endocrine Reviews*.

[35] Akinci A., Copeland K. C., Garmong A., Clemmons D. R. (2000). Insulin-like growth factor binding proteins (IGFBPs) in serum and urine and IGFBP-2 protease activity in patients with insulin-dependent diabetes mellitus. *Metabolism: Clinical and Experimental*.

[36] Feng X., Huang D., Lu X. (2014). Insulin-like growth factor 1 can promote proliferation and osteogenic differentiation of human dental pulp stem cells via mTOR pathway. *Development Growth and Differentiation*.

[37] Hu L., Yang G., Hägg D. (2015). IGF1 promotes adipogenesis by a lineage bias of endogenous adipose stem/progenitor cells. *Stem Cells*.

[38] Zhou Q., Li B., Zhao J., Pan W., Xu J., Chen S. (2016). IGF-I induces adipose derived mesenchymal cell chondrogenic differentiation in vitro and enhances chondrogenesis in vivo. *In Vitro Cellular and Developmental Biology—Animal*.

[39] Trosan P., Javorkova E., Zajicova A. (2016). The supportive role of insulin-like growth factor-I in the differentiation of murine mesenchymal stem cells into corneal-like cells. *Stem Cells and Development*.

[40] Huat T. J., Khan A. A., Pati S., Mustafa Z., Abdullah J. M., Jaafar H. (2014). IGF-1 enhances cell proliferation and survival during early differentiation of mesenchymal stem cells to neural progenitor-like cells. *BMC Neuroscience*.

[41] Ayatollahi M., Soleimani M., Geramizadeh B., Imanieh M. H. (2011). Insulin-like growth factor 1 (IGF-I) improves hepatic differentiation of human bone marrow-derived mesenchymal stem cells. *Cell Biology International*.

[42] Butler A. A., Yakar S., Gewolb I. H., Karas M., Okubo Y., LeRoith D. (1998). Insulin-like growth factor-I receptor signal transduction: at the interface between physiology and cell biology. *Comparative Biochemistry and Physiology Part B: Biochemistry & Molecular Biology*.

[43] Rinderknecht E., Humbel R. E. (1978). The amino acid sequence of human insulin-like growth factor I and its structural homology with proinsulin. *Journal of Biological Chemistry*.

[44] Rubin R., Baserga R. (1995). Insulin-like growth factor-I receptor: its role in cell proliferation, apoptosis, and tumorigenicity. *Laboratory Investigation*.

[45] Olson T. S., Bamberger M. J., Lane M. D. (1988). Post-translational changes in tertiary and quaternary structure of the insulin proreceptor. Correlation with acquisition of function. *Journal of Biological Chemistry*.

[46] Frasca F., Pandini G., Scalia P. (1999). Insulin receptor isoform A, a newly recognized, high-affinity insulin- like growth factor II receptor in fetal and cancer cells. *Molecular and Cellular Biology*.

[47] Xinaris C., Morigi M., Benedetti V. (2013). A novel strategy to enhance mesenchymal stem cell migration capacity and promote tissue repair in an injury specific fashion. *Cell Transplantation*.

[48] Clemmons D. R. (1992). IGF binding proteins: regulation of cellular actions. *Growth Regulation*.

[49] Jones J. I., Clemmons D. R. (1995). Insulin-like growth factors and their binding proteins: biological actions. *Endocrine Reviews*.

[50] Kelley K. M., Oh Y., Gargosky S. E. (1996). Insulin-like growth factor-binding proteins (IGFBPs) and their regulatory dynamics. *International Journal of Biochemistry and Cell Biology*.

[51] Shimasaki S., Ling N. (1991). Identification and molecular characterization of insulin-like growth factor binding proteins (IGFBP-1, -2, -3, -4, -5 and -6). *Progress in Growth Factor Research*.

[52] Cohick W. S., Clemmons D. R. (1993). The insulin-like growth factors. *Annual Review of Physiology*.

[53] Grimberg A., Cohen P. (2000). Role of insulin-like growth factors and their binding proteins in growth control and carcinogenesis. *Journal of Cellular Physiology*.

[54] Bach L. A., Headey S. J., Norton R. S. (2005). IGF-binding proteins—the pieces are falling into place. *Trends in Endocrinology and Metabolism*.

[55] Baserga R., Hongo A., Rubini M., Prisco M., Valentinis B. (1997). The IGF-I receptor in cell growth, transformation and apoptosis. *Biochimica et Biophysica Acta*.

[56] Bach L. A., Hsieh S., Sakano K.-I., Fujiwara H., Perdue J. F., Rechler M. M. (1993). Binding of mutants of human insulin-like growth factor II to insulin-like growth factor binding proteins 1–6. *Journal of Biological Chemistry*.

[57] Benyoucef S., Surinya K. H., Hadaschik D., Siddle K. (2007). Characterization of insulin/IGF hybrid receptors: contributions of the insulin receptor L2 and Fn1 domains and the alternatively spliced exon 11 sequence to ligand binding and receptor activation. *Biochemical Journal*.

[58] Baserga R. (2009). The insulin receptor substrate-1: a biomarker for cancer?. *Experimental Cell Research*.

[59] Chambard J.-C., Lefloch R., Pouysségur J., Lenormand P. (2007). ERK implication in cell cycle regulation. *Biochimica et Biophysica Acta—Molecular Cell Research*.

[60] Zandstra P. W., Nagy A. (2001). Stem cell bioengineering. *Annual Review of Biomedical Engineering*.

[61] Forbes K., Westwood M. (2008). The IGF axis and placental function: a mini review. *Hormone Research*.

[62] Myers M. G., Grammer T. C., Wang L.-M. (1994). Insulin receptor substrate-1 mediates phosphatidylinositol 3′-kinase and p70S6k signaling during insulin, insulin-like growth factor-1, and interleukin-4 stimulation. *Journal of Biological Chemistry*.

[63] van der Geer P., Wiley S., Gish G. D. (1996). Identification of residues that control specific binding of the Shc phosphotyrosine-binding domain to phosphotyrosine sites. *Proceedings of the National Academy of Sciences of the United States of America*.

[64] Van Der Geer P., Wiley S., Gish G. D., Pawson T. (1996). The Shc adaptor protein is highly phosphorylated at conserved, twin tyrosine residues (Y239/240) that mediate protein-protein interactions. *Current Biology*.

[65] Blenis J. (1993). Signal transduction via the MAP kinases: proceed at your own RSK. *Proceedings of the National Academy of Sciences of the United States of America*.

[66] Margolis B., Skolnik E. Y. (1994). Activation of ras by receptor tyrosine kinases. *Journal of the American Society of Nephrology*.

[67] Lamothe B., Bucchini D., Jami J., Joshi R. L. (1995). Interaction of p85 subunit of PI 3-kinase with insulin and IGF-1 receptors analysed by using the two-hybrid system. *FEBS Letters*.

[68] Valentinis B., Baserga R. (2001). IGF-I receptor signalling in transformation and differentiation. *Molecular Pathology*.

[69] Esposito D. L., Li Y., Cama A., Quon M. J. (2001). Tyr^612^ and Tyr^632^ in human insulin receptor substrate-1 are important for full activation of insulin-stimulated phosphatidylinositol 3-kinase activity and translocation of GLUT4 in adipose cells. *Endocrinology*.

[70] Roberts C. T., Owens J. A., Carter A. M., Harding J. E., Austgulen R., Wlodek M. (2003). Insulin-like growth factors and foetal programming—a workshop report. *Placenta*.

[71] Liu J.-P., Baker J., Perkins A. S., Robertson E. J., Efstratiadis A. (1993). Mice carrying null mutations of the genes encoding insulin-like growth factor I (Igf-1) and type 1 IGF receptor (Igf1r). *Cell*.

[72] Wang J., Zhou J., Bondy C. A. (1999). Igf1 promotes longitudinal bone growth by insulin-like actions augmenting chondrocyte hypertrophy. *FASEB Journal*.

[73] Rodriguez-de la Rosa L., Fernandez-Sanchez L., Germain F. (2012). Age-related functional and structural retinal modifications in the Igf1-/- null mouse. *Neurobiology of Disease*.

[74] DeChiara T. M., Efstratiadis A., Robertsen E. J. (1990). A growth-deficiency phenotype in heterozygous mice carrying an insulin-like growth factor II gene disrupted by targeting. *Nature*.

[75] Butler A. A., LeRoith D. (2001). Tissue-specific versus generalized gene targeting of the IGF1 and IGF1R genes and their roles in insulin-like growth factor physiology. *Endocrinology*.

[76] Liu J.-L., Yakar S., Leroith D. (2000). Conditional knockout of mouse insulin-like growth factor-1 gene using the Cre/loxP system. *Proceedings of the Society for Experimental Biology and Medicine*.

[77] Sheng M. H.-C., Zhou X.-D., Bonewald L. F., Baylink D. J., Lau K.-H. W. (2013). Disruption of the insulin-like growth factor-1 gene in osteocytes impairs developmental bone growth in mice. *Bone*.

[78] De Magalhaes Filho C. D., Kappeler L., Dupont J. (2017). Deleting IGF-1 receptor from forebrain neurons confers neuroprotection during stroke and upregulates endocrine somatotropin. *Journal of Cerebral Blood Flow & Metabolism*.

[79] Chaker Z., Aïd S., Berry H., Holzenberger M. (2015). Suppression of IGF-I signals in neural stem cells enhances neurogenesis and olfactory function during aging. *Aging Cell*.

[80] Youssef A., Han V. K. M. (2016). Low oxygen tension modulates the insulin-like growth factor-1 or -2 signaling via both insulin-like growth factor-1 receptor and insulin receptor to maintain stem cell identity in placental mesenchymal stem cells. *Endocrinology*.

[81] Yamahara K., Harada K., Ohshima M. (2014). Comparison of angiogenic, cytoprotective, and immunosuppressive properties of human amnion- and chorion-derived mesenchymal stem cells. *PLoS ONE*.

[82] Sadat S., Gehmert S., Song Y.-H. (2007). The cardioprotective effect of mesenchymal stem cells is mediated by IGF-I and VEGF. *Biochemical and Biophysical Research Communications*.

[83] Xing L., Cui R., Peng L. (2014). Mesenchymal stem cells, not conditioned medium, contribute to kidney repair after ischemia-reperfusion injury. *Stem Cell Research and Therapy*.

[84] Hu C., Wu Y., Wan Y., Wang Q., Song J. (2008). Introduction of hIGF-1 gene into bone marrow stromal cells and its effects on the cell's biological behaviors. *Cell Transplantation*.

[85] Stewart M. H., Bendall S. C., Bhatia M. (2008). Deconstructing human embryonic stem cell cultures: niche regulation of self-renewal and pluripotency. *Journal of Molecular Medicine*.

[86] Park S.-B., Yu K.-R., Jung J.-W. (2009). BFGF enhances the IGFs-mediated pluripotent and differentiation potentials in multipotent stem cells. *Growth Factors*.

[87] Youssef A., Iosef C., Han V. K. M. (2014). Low-oxygen tension and IGF-I promote proliferation and multipotency of placental mesenchymal stem cells (PMSCs) from different gestations via distinct signaling pathways. *Endocrinology*.

[88] Montes R., Ligero G., Sanchez L. (2009). Feeder-free maintenance of hESCs in mesenchymal stem cell-conditioned media: distinct requirements for TGF-beta and IGF-II. *Cell Research*.

[89] Lee H., Chang H., Lee S. (2016). Role of IGF1R^+^ MSCs in modulating neuroplasticity via CXCR4 cross-interaction. *Scientific Reports*.

[90] Ziegler A. N., Schneider J. S., Qin M. (2012). IGF-II promotes stemness of neural restricted precursors. *Stem Cells*.

[91] Ziegler A. N., Chidambaram S., Forbes B. E., Wood T. L., Levison S. W. (2014). Insulin-like growth factor-II (IGF-II) and IGF-II Analogs with enhanced insulin receptor-a binding affinity promote neural stem cell expansion. *Journal of Biological Chemistry*.

[92] Ziegler A. N., Levison S. W., Wood T. L. (2015). Insulin and IGF receptor signalling in neural-stem-cell homeostasis. *Nature Reviews Endocrinology*.

[93] Sancho-Martinez I., Baek S. H., Izpisua Belmonte J. C. (2012). Lineage conversion methodologies meet the reprogramming toolbox. *Nature Cell Biology*.

[94] Lee J. Y., Zhou Z., Taub P. J. (2011). BMP-12 treatment of adult mesenchymal stem cells in vitro augments tendon-like tissue formation and defect repair in vivo. *PLoS ONE*.

[95] De Coppi P., Bartsch G., Siddiqui M. M. (2007). Isolation of amniotic stem cell lines with potential for therapy. *Nature Biotechnology*.

[96] Wang N., Zhang R., Wang S.-J. (2013). Vascular endothelial growth factor stimulates endothelial differentiation from mesenchymal stem cells via Rho/myocardin-related transcription factor—a signaling pathway. *International Journal of Biochemistry and Cell Biology*.

[97] Oswald J., Boxberger S., Jørgensen B. (2004). Mesenchymal stem cells can be differentiated into endothelial cells in vitro. *Stem Cells*.

[98] de la Garza-Rodea A. S., van der Velde-van Dijke I., Boersma H. (2012). Myogenic properties of human mesenchymal stem cells derived from three different sources. *Cell Transplantation*.

[99] Zheng Y.-H., Xiong W., Su K., Kuang S.-J., Zhang Z.-G. (2013). Multilineage differentiation of human bone marrow mesenchymal stem cells in vitro and in vivo. *Experimental and Therapeutic Medicine*.

[100] Katagiri T., Takahashi N. (2002). Regulatory mechanisms of osteoblast and osteoclast differentiation. *Oral Diseases*.

[101] Mohan S., Baylink D. J. (1991). Bone growth factors. *Clinical Orthopaedics and Related Research*.

[102] Niu T., Rosen C. J. (2005). The insulin-like growth factor-I gene and osteoporosis: a critical appraisal. *Gene*.

[103] Clemens T. L., Chernausek S. D. (2004). Genetic strategies for elucidating insulin-like growth factor action in bone. *Growth Hormone & IGF Research*.

[104] Perrini S., Natalicchio A., Laviola L. (2008). Abnormalities of insulin-like growth factor-I signaling and impaired cell proliferation in osteoblasts from subjects with osteoporosis. *Endocrinology*.

[105] Koch H., Jadlowiec J. A., Campbell P. G. (2005). Insulin-like growth factor-I induces early osteoblast gene expression in human mesenchymal stem cells. *Stem Cells and Development*.

[106] Katagiri W., Osugi M., Kawai T., Hibi H. (2016). First-in-human study and clinical case reports of the alveolar bone regeneration with the secretome from human mesenchymal stem cells. *Head and Face Medicine*.

[107] Fanganiello R. D., Ishiy F. A. A., Kobayashi G. S., Alvizi L., Sunaga D. Y., Passos-Bueno M. R. (2015). Increased in vitro osteopotential in SHED associated with higher IGF2 expression when compared with hASCs. *Stem Cell Reviews and Reports*.

[108] Miller A. G., Aplin J. D., Westwood M. (2005). Adenovirally mediated expression of insulin-like growth factors enhances the function of first trimester placental fibroblasts. *The Journal of Clinical Endocrinology and Metabolism*.

[109] Adams T. E., Epa V. C., Garrett T. P. J., Ward C. W. (2000). Structure and function of the type 1 insulin-like growth factor receptor. *Cellular and Molecular Life Sciences*.

[110] Wilson E. M., Rotwein P. (2006). Control of MyoD function during initiation of muscle differentiation by an autocrine signaling pathway activated by insulin-like growth factor-II. *Journal of Biological Chemistry*.

[111] Putzer P., Breuer P., Götz W. (1998). Mouse insulin-like growth factor binding protein-6: expression, purification, characterization and histochemical localization. *Molecular and Cellular Endocrinology*.

[112] Schneider M. R., Lahm H., Wu M., Hoeflich A., Wolf E. (2000). Transgenic mouse models for studying the functions of insulin-like growth factor-binding proteins. *FASEB Journal*.

[113] Pumberger M., Qazi T. H., Ehrentraut M. C. (2016). Synthetic niche to modulate regenerative potential of MSCs and enhance skeletal muscle regeneration. *Biomaterials*.

[114] Zhang G.-W., Gu T.-X., Guan X.-Y. (2015). HGF and IGF-1 promote protective effects of allogeneic BMSC transplantation in rabbit model of acute myocardial infarction. *Cell Proliferation*.

[115] Guo J., Zheng D., Li W.-F., Li H.-R., Zhang A.-D., Li Z.-C. (2014). Insulin-like growth factor 1 treatment of MSCs attenuates inflammation and cardiac dysfunction following MI. *Inflammation*.

[116] Hsiao S. T.-F., Asgari A., Lokmic Z. (2012). Comparative analysis of paracrine factor expression in human adult mesenchymal stem cells derived from bone marrow, adipose, and dermal tissue. *Stem Cells and Development*.

[117] Fritton J. C., Kawashima Y., Mejia W. (2010). The insulin-like growth factor-1 binding protein acid-labile subunit alters mesenchymal stromal cell fate. *Journal of Biological Chemistry*.

[118] Tao Y., Zhou X., Liang C. (2015). TGF- *β* 3 and IGF-1 synergy ameliorates nucleus pulposus mesenchymal stem cell differentiation towards the nucleus pulposus cell type through MAPK/ERK signaling. *Growth Factors*.

[119] Longobardi L., O'Rear L., Aakula S. (2006). Effect of IGF-I in the chondrogenesis of bone marrow mesenchymal stem cells in the presence or absence of TGF-*β* signaling. *Journal of Bone and Mineral Research*.

[120] Longobardi L., Granero-Moltó F., O'Rear L. (2009). Subcellular localization of IRS-1 in IGF-I-mediated chondrogenic proliferation, differentiation and hypertrophy of bone marrow mesenchymal stem cells. *Growth Factors*.

[121] Liu W., D'Ercole J. A., Ye P. (2011). Blunting type 1 insulin-like growth factor receptor expression exacerbates neuronal apoptosis following hypoxic/ischemic injury. *BMC Neuroscience*.

[122] Park S. H., Kim K. W., Kim J. C. (2015). The role of insulin-like growth factor binding protein 2 (IGFBP2) in the regulation of corneal fibroblast differentiation. *Investigative Ophthalmology & Visual Science*.

[123] Zhao L., Feng Y., Chen X. (2016). Effects of IGF-1 on neural differentiation of human umbilical cord derived mesenchymal stem cells. *Life Sciences*.

[124] Semenova E., Koegel H., Hasse S. (2008). Overexpression of mIGF-1 in keratinocytes improves wound healing and accelerates hair follicle formation and cycling in mice. *American Journal of Pathology*.

[125] Edmondson S. R., Thumiger S. P., Werther G. A., Wraight C. J. (2003). Epidermal homeostasis: the role of the growth hormone and insulin-like growth factor systems. *Endocrine Reviews*.

[126] Todorović V., Peško P., Micev M. (2008). Insulin-like growth factor-I in wound healing of rat skin. *Regulatory Peptides*.

[127] Kato J., Kamiya H., Himeno T. (2014). Mesenchymal stem cells ameliorate impaired wound healing through enhancing keratinocyte functions in diabetic foot ulcerations on the plantar skin of rats. *Journal of Diabetes and Its Complications*.

[128] Zhou Y., Hu Q., Chen F. (2015). Human umbilical cord matrix-Derived stem cells exert trophic effects on *β*-cell survival in diabetic rats and isolated islets. *DMM Disease Models and Mechanisms*.

[129] Spitzer T. L. B., Rojas A., Zelenko Z. (2012). Perivascular human endometrial mesenchymal stem cells express pathways relevant to self-renewal, lineage specification, and functional phenotype. *Biology of Reproduction*.

[130] Saegusa J., Yamaji S., Ieguchi K. (2009). The direct binding of insulin-like growth factor-1 (IGF-1) to integrin *α*v*β*3 is involved in IGF-1 signaling. *The Journal of Biological Chemistry*.

[131] Ye P., Hu Q., Liu H., Yan Y., D'Ercole A. J. (2010). *β*-catenin mediates insulin-like growth factor-I actions to promote cyclin D1 mRNA expression, cell proliferation and survival in oligodendroglial cultures. *GLIA*.

[132] Huang Y.-L., Qiu R.-F., Mai W.-Y. (2012). Effects of insulin-like growth factor-1 on the properties of mesenchymal stem cells in vitro. *Journal of Zhejiang University: Science B*.

[133] Li Y., Yu X., Lin S., Li X., Zhang S., Song Y.-H. (2007). Insulin-like growth factor 1 enhances the migratory capacity of mesenchymal stem cells. *Biochemical and Biophysical Research Communications*.

[134] Chen L., Jiang W., Huang J. (2010). Insulin-like growth factor 2 (IGF-2) potentiates BMP-9-induced osteogenic differentiation and bone formation. *Journal of Bone and Mineral Research*.

[135] Shi Y., Chen J., Karner C. M., Long F. (2015). Hedgehog signaling activates a positive feedback mechanism involving insulin-like growth factors to induce osteoblast differentiation. *Proceedings of the National Academy of Sciences of the United States of America*.

[136] Madhala-Levy D., Williams V. C., Hughes S. M., Reshef R., Halevy O. (2012). Cooperation between Shh and IGF-I in promoting myogenic proliferation and differentiation via the MAPK/ERK and PI3K/Akt pathways requires smo activity. *Journal of Cellular Physiology*.

[137] Li L., Neaves W. B. (2006). Normal stem cells and cancer stem cells: the niche matters. *Cancer Research*.

[138] Hsieh A., Ellsworth R., Hsieh D. (2011). Hedgehog/GLI1 regulates IGF dependent malignant behaviors in glioma stem cells. *Journal of Cellular Physiology*.

[139] Xu C., Xie D., Yu S.-C. (2013). *β*-catenin/POU5F1/SOX2 transcription factor complex mediates IGF-I receptor signaling and predicts poor prognosis in lung adenocarcinoma. *Cancer Research*.

[140] Fiore E. J., Bayo J. M., Garcia M. G. (2015). Mesenchymal stromal cells engineered to produce IGF-I by recombinant adenovirus ameliorate liver fibrosis in mice. *Stem Cells and Development*.

[141] Winter E. M., Van Oorschot A. A. M., Hogers B. (2009). A new direction for cardiac regeneration therapy: application of synergistically acting epicardium-derived cells and cardiomyocyte progenitor cells. *Circulation: Heart Failure*.

[142] Kearns-Jonker M., Dai W., Gunthart M. (2012). Genetically engineered mesenchymal stem cells influence gene expression in donor cardiomyocytes and the recipient heart. *Journal of Stem Cell Research & Therapy*.

[143] Gao D., Xie J., Zhang J. (2014). MSC attenuate diabetes-induced functional impairment in adipocytes via secretion of insulin-like growth factor-1. *Biochemical and Biophysical Research Communications*.

[144] Yulyana Y., Ho I. A. W., Sia K. C. (2015). Paracrine factors of human fetal MSCs inhibit liver cancer growth through reduced activation of IGF-1R/PI3K/Akt signaling. *Molecular Therapy*.

[145] Kuhn C., Hurwitz S. A., Kumar M. G., Cotton J., Spandau D. F. (1999). Activation of the insulin-like growth factor-1 receptor promotes the survival of human keratinocytes following ultraviolet B irradiation. *International Journal of Cancer*.

